# 1398. Gland Tuberculosis: A Rare Localization of Tuberculosis

**DOI:** 10.1093/ofid/ofab466.1590

**Published:** 2021-12-04

**Authors:** Fatma Hammami, Makram Koubaa, Amal Chakroun, Khaoula Rekik, Chakib Marrakchi, Fatma Smaoui, Mounir Ben Jemaa

**Affiliations:** Infectious Diseases Department, Hedi Chaker University Hospital, University of Sfax, Tunisia, Sfax, Sfax, Tunisia

## Abstract

**Background:**

Tuberculosis (TB) is a multisystem disease that might affect all organs. Gland TB is characterized with a misleading clinical presentation which often mimic a neoplastic process. The aim of our work was to study the clinical, therapeutic and evolutionary features of gland TB.

**Methods:**

We conducted a retrospective study including all patients hospitalized for gland TB in the infectious disease department between 1999 and 2020.

**Results:**

We encountered 28 cases among which 24 were females (85.7%). The mean age was 39±14 years. A rural origin was noted in 15 cases (53.5%). Two patients were previously treated for TB (7.1%). Systemic symptoms of TB included fever (60.7%), asthenia (53.5%), loss of appetite (46.4%) and weight loss (25%). There were 18 cases of breast TB (64.3%), 4 cases of salivary gland TB (14.3%) and 3 cases of ovarian TB (10.7%). Two cases of pituitary TB (7.1%) and one case of adrenal TB (3.6%) were noted. Multifocal TB was noted in 7 cases (25%). Lymph node (17.8%), pulmonary (14.2%) and peritoneal (7.1%) TB were associated with gland TB. Tuberculin skin test was positive in 19 cases (67.8%). The diagnosis was based on histopathological proof in 23 cases (82.1%), microbiological proof in 4 cases (14.3%) and clinically confirmed in one case (3.6%). The median duration of antitubercular therapy was 10 [9-15] months. Patients received fixed-dose combination in 11 cases (39.2%). Adverse effects of antitubercular therapy were noted in 10 cases (35.7%) represented by gastrointestinal symptoms (14.3%), increase in hepatic enzyme levels (14.3%) and skin reactions (7.1%). The disease evolution was favorable in 26 cases (92.9%). Relapse was noted in two cases (7.1%).

**Conclusion:**

Gland TB included different sites. The presence of systemic symptoms of TB and the diagnosis of TB elsewhere in the body helped through the diagnosis process which requires high index of suspicion. It was mainly based on histological evidence.

**Disclosures:**

**All Authors**: No reported disclosures

